# Open drug scenes across city sizes: Socioeconomic status, crime patterns and community perspectives

**DOI:** 10.1177/14550725251327516

**Published:** 2025-03-17

**Authors:** Alberto P. Chrysoulakis, Manne Gerell, Mia-Maria Magnusson

**Affiliations:** Department of Criminology, 59606 Malmö universitet Fakulteten för hälsa och samhälle, Malmo, Sweden; Department of Criminology, 59606 Malmö universitet Fakulteten för hälsa och samhälle, Malmo, Sweden; 7664 Swedish Police Authority, Stockholm, Stockholm, Sweden

**Keywords:** crime, disorder, narcotics, open drug scenes, spatial analysis

## Abstract

**Aims:** Open drug scenes (ODS) have increasingly drawn the attention of the police and municipalities in Sweden. These locations, where illicit drugs are sold and/or consumed, are often associated with various forms of disorder and crime. While ODS are typically depicted as a phenomenon predominantly found in larger cities, their prevalence and characteristics in smaller cities remain underexplored. This study aims to analyse the patterns and characteristics of ODS, as identified by the police and municipalities, across a range of cities in southern Sweden. **Methods:** By utilising spatial and temporal analyses of police-reported crimes and demographic statistics, this research examines the characteristics of identified ODS and their connections to socioeconomic disadvantage. **Results:** The findings suggest that the identified ODS in smaller cities share similar patterns to those found in prior research and in larger urban areas, characterised by lower socioeconomic status and elevated crime rates. **Conclusions:** Police and municipalities in smaller cities identify places in their communities that closely resemble, although are not necessarily equivalent to, an ODS. Nevertheless, these places are disproportionately burdened by social problems and require targeted assistance.

## Introduction

Open drug scenes (ODS) in Sweden have increasingly drawn attention from the police and municipalities of various sizes because they are expected to enhance their crime prevention efforts in response to these sites. ODS are spatially defined places where narcotics are sold and used, potentially sustaining or increasing substance use and heightening the risk of adolescents becoming involved in trade and/or drug use (Chami et al., 2013; Fast et al., 2010; Hunt-Howard, 2017; Roy et al., 2008). Traditionally considered an urban issue, ODS are predominantly associated with larger cities. These sites frequently coincide with other forms of crime, such as (gun) violence (Gerell et al., 2021; Lum, 2008; Magnusson, 2023; Martínez et al., 2008; Torres et al., 2021) and various social disorders (Saberi Zafarghandi et al., 2022), especially in areas with lower socioeconomic status (SES) (Magnusson, 2023). However, there is a lack of research into ODS in smaller cities, despite evidence that drug markets extend beyond large urban places (c.f. working “county lines”; Harding, 2020; Robinson et al., 2019). This gap raises questions about the nature of ODS in smaller cities or communities.

When police and municipalities direct their attention towards ODS, discrepancies can arise between what these practitioners identify to be an ODS in their community and how these are defined in academic literature. The places identified and targeted for intervention by practitioners are crucial to study because it is where resources are allocated and where potential impacts on crime and disorder can be realised.

The aim of the present study is to address the knowledge gap regarding the extent to which identified ODS in smaller cities face challenges similar to those in larger cities. This study contributes to both theory and practice. Theoretically, it explores whether prior knowledge about ODS in urban settings applies to those identified in less-researched areas or whether alternative models of explanation are required. Practically, it addresses the characteristics of places identified by practitioners and the implications for crime prevention strategies.

### Background

Place-based drug markets have been documented in numerous locations across the globe. Despite the diversity of definitions and terminology used to describe them, the problems associated with these sites appear to be broadly shared, as are the efforts to counteract them. Local communities often face challenges related to drug activity and violence in these areas. While most studies and descriptions focus on cities or suburban areas, several actors in Sweden, including the police, media and the public (Berger et al., 2021; Brå, 2021; Smålandsposten, 2023), have highlighted that smaller cities and rural areas also experience similar issues. This is an under-researched aspect of the field.

At the same time, municipalities of all sizes are expected to increase their crime prevention efforts in response to these locations. As of 1 July 2023, Swedish legislation mandates that municipalities take on crime prevention responsibilites (SFS 2023:196, Lag om kommuners ansvar för brottsförebyggande arbete). In short, this law requires municipalities to implement crime prevention efforts based on local problem analyses and to coordinate these efforts with relevant stakeholders. A key part of these local analyses involves defining the specific problems to be addressed. In recent years, ODS have increasingly been included among the issues that police and municipalities of various sizes must tackle. These practitioners are assumed to identify places characterised as an ODS (more on definitions below) and especially for smaller cities it presents particular challenges. Do they have an ODS in their community (in an ontological sense)? If so, how do they identify the ODS (in an epistemological sense)? Or do they instead identify the places that most closely resemble an ODS (which need not be equivalent to an ODS)? This is part of the practitioner's operative reality and are important questions because it is the practitioners who ultimately make an impact at these places. What, then, are the characteristics of the ODS identified by practitioners?

Before describing the project on which this study is based, a summary is provided of existing knowledge on ODS from various perspectives and this is organised into three parts: definitions and characteristics of ODS, reasons for their emergence, and issues associated with them. It is important to note that while the harms connected to the use of illicit drugs and interventions to reduce such harm (Amundsen et al., 2024) may overlap with ODS, this study specifically focuses on ODS, rather than the broader issue of drug-related harm.

### Definition and characteristics of ODS

Internationally, the phenomena of drug-related issues in public spaces have been described with terms such as “open-air drug markets” (Corsaro et al., 2013), “street drug scene” (Aitken et al., 2002), “street(-level) drug market” (Coomber & Maher, 2006), “drug hot spots” (Weisburd & Mazerolle, 2000) or “open drug scene” (i.e., ODS) (Magnusson, 2020). An ODS represents a specific type of illicit drug market or venue. The term “illicit drug market” broadly encompasses various retail venues, such as street-based markets, online drug distribution, or other open, semi-open or closed markets (May & Houghy, 2004), sometimes focusing on specific substances rather than their accessibility (Degenhardt et al., 2004; Jacobs, 1999).

ODS are typically described as street-based open drug markets that are visible and accessible to the public. Earlier work has, for example, defined an ODS as “public areas where illegal hard drugs […] are being consumed and sold and which are tolerated by the police and political actors” (Eisner, 1993). Or more broadly, “all situations where citizens are publicly confronted with drug use and drug dealing” (Bless et al., 1995). In the Swedish context, the most commonly used definition comes from Magnusson (2020), who describes an ODS as “a geographical area, sustained in time and space, where use and dealing of drugs takes place in the public and is perceived as problematic by authorities and/or the public”. This definition has been used since 2017, beginning with a police project in the capital region of Stockholm, Sweden (for a thorough description, see Magnusson, 2020; Regional Police Stockholm, 2023, 2024). Today, this large collaborative project between the police and municipalities is based on research and has spread to other parts of the country. The Swedish police now have a national mapping tool for ODS, used by several other regions (www.oppnadrogscener.se). The definition builds on the knowledge from practitioners within authorities (primarily the police) and the civil society, enabling them to pinpoint these areas. ODS, as a phenomenon, is not easily captured by crime statistics or population data but instead is seen as a problem recognised by the police, municipalities and people living or working in these places (Magnusson, 2020; Regional Police Stockholm, 2024).

Questions concerning definitions are important because the definition used can influence which places are identified as ODS. Variations in definitions could also point towards why scholars have seen as variations in *types* of ODS. Bless et al. (1995) identified three ODS types across nine European cities based on visibility, size and site. Namely, (i) concentrated open scenes, (ii) dispersed open scenes and (iii) hidden scenes. Similarly, Magnusson (2020) identified three types within Stockholm, Sweden: (i) city ODS, (ii) vulnerable suburb ODS and (iii) suburb ODS. These typologies, while distinct, overlap in that both studies recognise the significance of transportation nodes, local and city centres, and residential areas.

Despite variations in labels, definitions and types, the core theme remains the illicit drug dealing and/or consumption that takes place in publicly accessible, spatially defined areas. This study adopts the Swedish police definition of ODS, as outlined above.

### The emergence of ODS

The emergence of ODS can be examined from two perspectives: their presence in larger communities (*why in some neighbourhoods but not others*?) and specific locations (*why at this particular place*?).

#### Why certain neighbourhoods?

ODS typically arise in socioeconomically disadvantaged areas or places frequented by people, such as transit nodes or local and city centres (Bless et al., 1995; Magnusson, 2020).

In disadvantaged neighbourhoods, the explanation often involves reduced informal social control, a concept rooted in Shaw and McKay's (1942) *social disorganisation theory.* This theory, further refined by Sampson et al. (1997) through the notion of *collective efficacy*, emphasises how the structural factors poverty, residential instability and racial/ethnic heterogeneity undermine social cohesion and informal social control, and, ultimately, the community's capacity to address disorder or crime. Consequently, such neighbourhoods exhibit lower collective efficacy, making them susceptible to the development of ODS.

Conversely, ODS in local and city centres are characterised by their mixed land use and transient populations rather than deprivation *per se* (Magnusson, 2020). The lack of (informal) social control is important for understanding crime rates at these places as well. At the same time, it points towards how ODS can occupy smaller spatial areas than the larger “neighbourhood”.

#### Why some places?

While certain neighbourhoods have more suitable conditions for ODS to emergence, generally, a confined part of it is deemed an ODS. Research on crime concentration suggests that, even within high-crime neighbourhoods, there can be places that remain crime-free over time (Groff & Weisburd, 2010). This raises the question: why do ODS emerge at some specific locations and not others? The *crime pattern theory* provides insight, suggesting that some places could be viewed as *crime generators* or *crime attractors*, offering opportunities for illicit activities like drug dealing or consumption (Brantingham & Brantingham, 1995)*.* Something with the built environment might attract congregation at the specific place. For example, factors such as proximity to public transportation enabling easy access, shelter and/or access to public restrooms. These places often lack ownership or guardianship, reducing the likelihood of intervention against disorder or crime.

### Problems associated with ODS

Beyond drug dealing and public drug use, ODS are linked to a variety of negative consequences. In their scoping review, Saberi Zafarghandi et al. (2022) identified common issues like drug related litter and loitering, other forms of crime, and sex work. The latter has also been found in ODS's in Stockholm, Sweden (Stallwitz, 2019). Violence is also prevalent in ODS, although it is not a consistent pattern across all contexts. Studies from various countries have noted connections between ODS and violence (Gerell et al., 2021; Hautala et al., 2019; Magnusson, 2020; Martínez et al., 2008; McNeil et al., 2014; Stallwitz, 2019). Ousey and Lee (2002, 2007), studying more than hundred US cities, found that changes in (gun-related) homicide rates followed changes in drug-market activity. As the latter expanded, so did homicide rates. However, they also point out that the parallel changes do not uniformly apply across different social contexts. Also, Lum (2008) stresses variation in the identified relationship between clusters of drug activities and violence. This could be why other scholars have not found a connection between ODS and violence (Eshrati et al., 2023; Thane, 2002). For this reason, the present study incorporates police reported narcotic offences as well as other types of crimes committed in public.

While most of the literature focuses on ODS as a negative phenomenon, they can have some positive influence as well; for example, as a meeting place where drug related problems can be discussed in a non-judgemental way (Grønnestad & Lalander, 2015).

### Aims

This study aims to analyse the patterns and characteristics of identified ODS across cities and communities of varying sizes in southern Sweden. Specifically, it addresses the following questions:

1. What are the characteristics, similarities and differences of identified ODS in southern Sweden?
2. How are these ODS characterised in terms of SES and crime statistics?

Police-reported offences and demographic information are used to explore these questions, focusing on ODS identified by practitioners in southern Sweden.

## Methods

### Project and study area

The present study is part of a larger project aimed at intervening and dissolving ODS. The County Administrative Board of Skåne (Länsstyrelsen Skåne) oversees the project, which involves six municipalities in southern Sweden, their local police force, the regional public transport service (Skånetrafiken) and Malmö university.

The municipalities were selected following a request from the County Administrative Board, with the following criteria: (i) an established collaboration agreement between the municipality and the police, (ii) the identification of one or more ODS for intervention and (iii) the commitment to the project for a minimum of 12 months. The project involves six municipalities and their respective police forces in southern Sweden. It is guided by the SARA model (Scanning, Analysis, Response and Assessment; see for example, Clarke & Eck, 2016; Eck & Spelman, 1987), which allows project participants to identify local problems through scanning and analysis, share experiences, develop tailored responses, and later assess the effectiveness of these interventions.

In addition to the ODS targeted within these municipalities, additional ODS in the region were selected as control sites. These control sites were identified by local police in other municipalities not participating in the project. Further details about these additional ODS are provided later in the Methods section.

The identification of each ODS was made by practitioners, primarily by the police, guided by the definition outlined earlier: a geographical area, sustained in time and space, where use and dealing of drugs takes place in the public and is perceived as problematic by authorities and/or the public. The sources of information to identify such places are primarily based on crime statistics, intelligence and expert knowledge.

In total, 16 ODS across ten municipalities were identified: eight from the project and eight matched control sites, chosen for their similarity in characteristics (i.e., whether the ODS is located near a public transportation node, in a park, in a residential neighbourhood, etc). Some ODS were, however, close in proximity and thus analysed together, rendering a total of 14 ODS analysed in this study.

**Figure 1.**
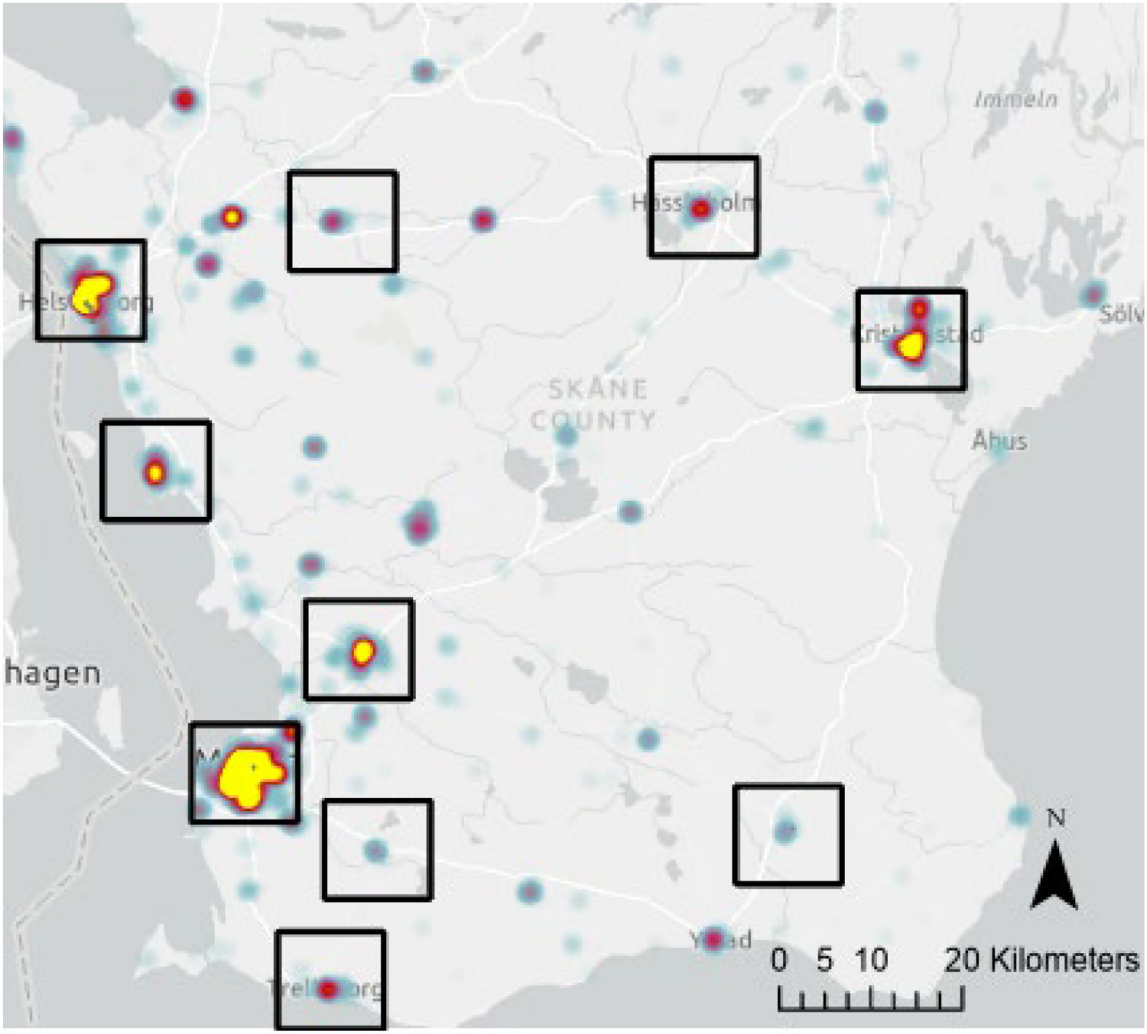
Heat map of reported narcotic offences in Skåne county. The black squares highlight cities with open drug scenes (ODS) included in this study.

Figure 1 depicts the selected cities’ locations, displaying a distribution across southern Sweden. Each location is highlighted through a kernel density estimation of police reported narcotics offences, which shows that the police are active at these locations. Bigger cities have more police officers and stronger concentrations of police reported drug crimes, but all the ODS in this study have elevated levels of police reported drug crimes to some extent.

### Official statistics

This study employs police-reported crimes and demographic statistics to explore the ODS. The former characterises the places in terms of crime and the second provides a theoretical backdrop in terms of SES.

#### Crime statistics

Two aspects of scanning the problem at each location are the spatial and temporal patterns of crime, assessing whether drug-related activities are isolated or part of broader issues, and their persistence over time.

The crime types analysed include assault outdoors, robbery, vandalism, theft, traffic offences and other specific criminal offences (such as shootings), in addition to dealing, possession and use of narcotics. It should be noted that narcotics and traffic offenses are often argued to mirror where the police focus their resources and should therefore not be seen as a reliable proxy for the amount of narcotics or traffic crime at a location. However, there are clear connections to other forms of crime as research has recently found that drug crimes are reported in relation to other crimes, such as stolen goods, thefts, violent crimes and possession of illegal weapons (mainly knifes), in between 40% (Egnell, 2023) and 75% (Ceccato et al., 2023) of cases in Sweden.

#### Demographic variables

The other source of official statistics concerns demography provided by Statistics Sweden (https://www.scb.se), including rates of unemployment, foreign-born residents and single-parent households as of 2021. These measures are part of a broader scale used in prior research to study living conditions (a form of SES), which in turn is used as a proxy for, or tapping into, theoretical notions of social disorganisation, collective efficacy and social stress (Gerell et al., 2022; Guldåker et al., 2021).

The data are aggregated to grids (250 × 250 m) across the densely populated parts of the region, totalling 13,801 grids. Each grid cell includes rates for each demographic variable. This fine-grained spatial analysis, based on grid cells rather than larger areas like neighborhoods, allows for more detailed insights.

Following Guldåker et al. (2021), the three demographic variables were combined into a single scale where each variable was categorised into five equally sized groups, with the exceptions for zero-value cells. All grid cells with value zero (i.e., no unemployed, foreign-born resident or single-parent household) were grouped separately. Table 1 presents the cut-offs, group distribution and number of cells in each group.

**Table 1.** Distribution of socioeconomic status predictors.

Group value	Proportion interval, number of grid cells		
	Non-employed	Foreign-born	Single parent
1	0, *n* = 3449	0, *n* = 4844	0, *n* = 8314
2	0.027–0.155, *n* = 2316	0.012–0.095, *n* = 2133	0.007–0.067, *n* = 1253
3	0.156–0.239, *n* = 2334	0.096–0.160, *n* = 2130	0.068–0.094, *n* = 1275
4	0.240–0.400, *n* = 2323	0.161–0.275, *n* = 2135	0.095–0.137, *n* = 1267
5	0.401–1, *n* = 2349	0.276–1, *n* = 2133	0.138–1, *n* = 1265
Mean	0.249	0.140	0.050
SD	0.282	0.183	0.108

The SES scale, ranging from 1–5, is derived by averaging the group values for each grid cell. Higher values indicate lower SES. Analyses excluded 1097 grids with missing data, focusing on the remaining 12,704 grids.

### Analytic strategy

This study is based on quantitative analyses of the official statistics. Quantitative data addresses the characterisation of ODS (research question (RQ) 2). The comparison of ODS and alignment with previous research addresses RQ1 regarding similarities and differences.

The quantitative analyses map spatial patterns of crime statistics together with the demographic information. It also explores temporal patterns seeing that ODS should be temporally stable. The main analyses are binary logistic regressions with grids as unit of analyses. The outcome, grid cells overlapping/intersecting with an ODS, is regressed on SES (model 1), on logarithmic transformed total count of crime and narcotic offences (to account for the skewness), respectively (model 2 and model 3) and all three predictors (model 4). The analyses were conducted in ArcGIS Pro 3.0.3 (https://www.esri.com/en-us/arcgis/products/arcgis-pro).

## Results

### Description of identified ODS

The identified ODS in this study exhibit both commonalities and differences in their characteristics. Drawing inspiration from Magnusson (2020), Table 2 summarises these characteristics along with descriptive statistics for each ODS.

**Table 2.** Descriptives of the ODS.

Place	City ODS	Vulnerable suburb ODS	Suburb ODS	Municipality population size, thousands, 2022	Size of the ODS (km^2^)	Number of all reported offences excluding narcotics, *n* = 3802 (within 100 m buffer, *n* = 8069), 2018–2021	Number of reported narcotic offences, *n* = 1921 (within 100 m buffer, *n* = 3336), 2018–2021
Helsingborg 1	X			152	0,12	810 (3244)	622 (1420)
Helsingborg 2		X		152	0,14	309 (478)	130 (153)
Helsingborg 3	X			152	0,08	429 (3244)	142 (1420)
Helsingborg 4		X		152	0,21	742 (3244)	243 (1420)
Hässleholm	X			52	0,05	204 (494)	73 (120)
Klippan	X			18	0,11	155 (360)	82 (153)
Kristianstad			X	87	0,05	18 (100)	7 (52)
Landskrona 1			X	47	0,05	73 (168)	47 (77)
Landskrona 2			X	47	0,11	324 (763)	94 (192)
Lund	X			128	0,02	102 (475)	28 (127)
Malmö	X			357	0,11	400 (1327)	415 (924)
Svedala	X			23	0,02	59 (132)	12 (25)
Tomelilla	X			14	0,05	123 (226)	17 (30)
Trelleborg	X			47	0,04	64 (312)	9 (73)
Mean				92,5	0,07	272,29 (673,25)	137,21 (278,83)
SD				103,88	0,06	250,79 (876,94)	178,90 (433,47)

Predominantly, the ODS are located in city centres with a comparatively high flow of people. Most are situated near a transportation node such as the central railway or bus station, although some are in residential areas without a central transportation node.

Table 2 (columns 4 and 5) illustrates how the ODS vary in size, both concerning the population of the municipality and the physical area of the ODS. While larger municipalities tend to have larger ODS, this correlation is not statistically significant at the 5% level of significance (rs = .51 (S = 224.66), p = 0.06).

Table 2 also presents data on all reported offences (assault outdoors, robbery, vandalism, theft, traffic offences, and other specific criminal offences such as shootings; column 7) and narcotic offences (column 8), both within the ODS and within a 100-m buffer zone. Generally, the data indicate two reported crimes for every narcotic offence across all ODS, suggesting that ODS are associated with a broader spectrum of criminal activities besides narcotics or the policing of narcotics. Only the largest city in the study, Malmö, presents an inverse ratio with more narcotic offences than other crimes.

Although the definition of an ODS suggests that the area is delineated by specific boundaries, human interactions and the dynamics of routine activities often extend beyond these limits. To account for this, 100-m buffer zones are included in Table 2, capturing adjacent areas. Comparing crimes reported within the ODS to those also reported within a 100-m buffer zone shows that most ODS have extensive criminal activity beyond the ODS boundaries. This raises the question of how to define the boundaries of an ODS, which is a question revisited in the Discussion.

### Crime statistics: temporal trends

**Figure 2.**
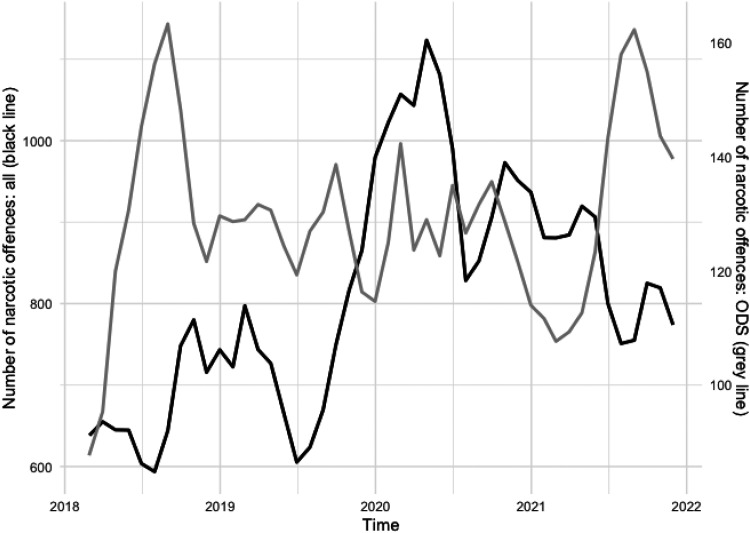
Development of number of reported narcotic offences, 3 months rolling average. Black line: all narcotic offences in the region corresponding to left *y*-axis. Grey line: all narcotic offences in the open drug scenes (ODS) corresponding to the right *y*-axis.

ODS typically show consistent drug-related problems making temporal trends an essential area of study. Results show that narcotic offences at ODS do not follow the same trend as narcotic offences in general. Figure 2 illustrates the development of eleven different types of narcotic offences from January 2018 to December 2022 (note the different axis values). The numbers are driven by the three offences *distribution*, *possession* and *use* of narcotics which together comprise 97% of the total 35,721 narcotic offences reported in the region during this period.

While the overall narcotic offences (black line) increased until mid-2020 before declining, offences at ODS (grey line) displayed a more stable trend with peaks in August 2018 and August 2021.

**Figure 3.**
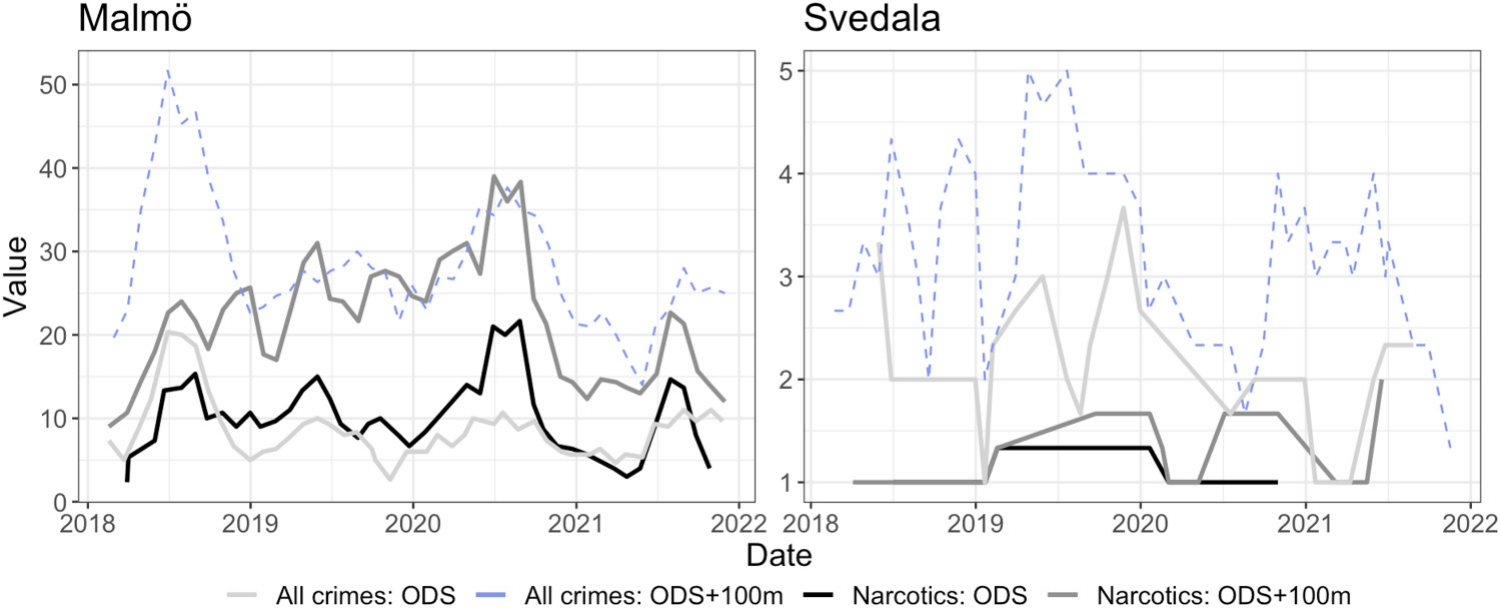
Temporal trends for reported crimes aggregated by months, 3 months rolling average. Largest (left) and smallest (right) municipality in the study.

However, the patterns vary in magnitude by location. Figure 3 contrasts the temporal trends in the largest and smallest municipality, illustrating particularly low number of narcotic offences in smaller municipalities. The number of reported narcotic offences at ODS in such smaller municipalities, and within their 100-m buffer zone, are consistently low. This likely reflects fewer police officers being available in smaller cities, and narcotics is a crime that typically is police initiated, meaning that, to a large extent, it reflects police resources, priorities and strategies (Lundgren, 2018).

### Crime statistics: spatial patterns and SES

Crime in general, and policing of narcotic offences in particular, tends to cluster and correlate with lower SES at an aggregate level (Lundgren, 2018). When it comes to SES, analyses show a clustering effect (Moran's *I* = 0*.*28, *p *< .001), meaning that lower SES grids often are connected to other grids with lower SES. Furthermore, the average SES is lower in grids connected to an ODS. In general, the mean value of SES in the 12,704 analysed grids is 2.49 (SD=1.01), compared to 3.96 (SD=0.62) in grids intersecting an ODS.

The amount of crimes at the identified ODS are highly disproportionate. Although grids intersecting with an ODS constitute only 0.5% of all grids (*n* = 64), they account for 5% (*n* = 9990) of all recorded crimes (*n* = 198,135) of the types analysed in this study, and 11% (*n* = 4335) of all narcotic offences (*n* = 38 692) during 2018–2021.

Thus, both SES and the occurrence of crime seem connected to the ODS. Statistical tests of these connections are displayed in Table 3, exploring the extent to which SES and crime counts predict ODS emergence. The results indicate that both factors are significant, with crime occurrence being a more potent predictor. Models 1–3 model the predictors SES, crime count (logged) and narcotic count (logged) separately to analyse their separate relationship to the presence of an ODS. Model 4 incorporates all three.

**Table 3.** Grids connected to an ODS (no/yes) regressed on the grids SES and the grids count of crime and narcotic offences (log transformed), respectively.

Variable	Coefficient (SE)	z-Statistic	Odds Ratio	Wald's Low-High (95%)	VIF	AIC	Deviance explained
Model 1							
Intercept	−10,57 (0,63)	−16,70*	0	0		659,787	0,174
SES	1,6 (0,16)	10,25*	4,98	3,66–6,76			
Model 2							
Intercept	−10,15 (0,55)	−18,39*	0	0		516,85	0,354
Crime count	1,54 (0,12)	12,98*	4,67	3,7–5,9			
Model 3							
Intercept	−7,61 (0,31)	−24,26*	0	0		508,738	0,365
Narcotics count	1,36 (0,09)	15,47*	3,89	3,27–4,61			
Model 4							
Intercept	−10,44 (0,84)	−12,49*	0	0	—	489,841	0,393
SES	0,56 (0,19)	2,90*	1,75	1,2–2,54	1,36		
Crime count	0,69 (0,18)	3,85*	2	1,4–2,84	2,22		
Narcotics count	0,67 (0,21)	3,19*	1,95	1,29–2,94	2,37		

Total number of grids is *n* = 12,704, grids connected to an ODS is *n* = 63. AIC = Akaike information criterion; SES = socioeconomic status; VIF = variance inflation factor.

*p < 0.01.

Model 1 shows that grids with lower SES increases the odds of a grid being classified as an ODS. That is, lower SES predicts the occurrence of an ODS. Similarly, model 2 shows that grids with a higher count of offences also increase the odds of a grid being classified as an ODS, which is also the case when controlling for narcotic offences (model 3). (The same is true when using a non-transformed total count of narcotic offences, as the odds ratio then is 1.031 (Wald's 95% confidence interval: low = 1.025, high = 1.036.) This indicates that the ODS identified in this study tend to be burdened by higher levels of crime in general, exhibiting clusters of crime as well as narcotic offences. The same is true when considering all three predictors together in model 4. The model emerges as the best fitting model with lowest Akaike information criterion value explaining most of the variance in the outcome. This suggests that both the volume of reported offences and SES are important in understanding the emergence of ODS. While there is considerable overlap between total and narcotic offences as predictors, the model highlights individual contributions to ODS classification. (Multicolinearity is not a cause of concern as no VIF value exceeds 2.5.) There might be ODS with considerable reported narcotic offences but not total offences and vice versa. However, the presence of autocorrelated residuals in the models suggests that other significant predictors have been omitted from the analyses.

## Discussion

The present study aimed to fill a gap in prior research concerning identified ODS in places beyond larger cities, focusing on their SES and crime prevalence. The findings show that much of what is known about ODS through prior research also applies to ODS in smaller cities. They share characteristics in terms of emerging where (i) relatively more people tend to spend time or pass through, such as public transportation nodes or parks and (ii) places with lower SES. As such, crime pattern theory (Brantingham & Brantingham, 1995) and collective efficacy (Sampson et al., 1997) offer valuable frameworks for explaining the phenomena also in smaller communities and guiding some forms of crime preventive efforts. Moreover, these locations are often burdened by a range of issues beyond drug-related activities, including various other forms of crime occurring in public spaces.

A notable distinction between ODS in smaller versus larger cities lies in their persistence. Prior research highlights the enduring nature of ODS in major urban areas (cf. “Plattan” in Stockholm, Sweden (Magnusson, 2020) or “Plata” in Oslo, Norway (Sandberg & Pedersen, 2008)), but endurance is not quite as established in smaller cities as illustrated by the temporal analyses. This inconsistency raises critical questions regarding the classification of certain areas as ODS. For example, whether the areas identified by the police and municipalities as ODS fall under the definition “a geographical area, sustained in time and space, where use and dealing of drugs takes place in the public and is perceived as problematic by authorities and/or the public”? Or are they simply places where drug dealing or consumption occasionally occurs, rather than established vending or consumption sites? Conflating occasional drug activity with a fully established ODS may lead to inaccurate classifications. It would be misleading to equate the established ODS in large cities with those in smaller communities, as demonstrated in this study.

This distinction becomes increasingly relevant with the recent legislative changes in Sweden, emphasising municipal responsibilities in crime prevention, underscoring the importance of accurate ODS identification to ensure effective resource allocation. Therefore, a thorough analysis is needed before defining a place as an ODS so as to not conflate it with a place where adolescents spend time unsupervised or perceived drug addicts congregate. At the same time, the results indicate that the places identified in smaller cities might be the places with the comparatively highest prevalence of drug-related activities thus being *their* equivalence of an ODS. Whether or not these areas meet the strict definition of an ODS, the places identified by practitioners are typically crime-burdened and exhibit patterns of lower SES, which is often used as a proxy for reduced (informal) social control.

As such, these findings underscore the need for crime preventive measures that extend beyond street-level drug policing, which has traditionally been a default response to the issue in Nordic countries (Laursen, 1995). The project on which this study is based illustrates a more comprehensive approach, fostering cooperation among practitioners with different expertise, responsibilities and toolkits, thus creating a broader infrastructure for addressing these complex issues.

### Limitations

Mapping out ODS using different sources, as done in the present study, is a strength offering diverse perspectives. At the same time, the methods are not without its limitations. Most notably, they concern (i) the delineation of ODS boundaries and (ii) reliance on crime statistics.

Defining the spatial extent of an ODS presents challenges, as demonstrated by the significant crime reporting within a 100-m buffer zone outside designated ODS boundaries. It means that drawing the boundary in a slightly different way could affect the results. This issue, known as the *modifiable areal unit problem,* highlights the trade-off between accurately representing the problem area and maintaining spatial specificity (Gerell, 2017).

Another problem with drawing spatial boundaries is connected to crime statistics. There is a difference between where the crime was committed and where the police reported it. For car arson, the median error is 83 m (Gerell, 2018). To what extent narcotic offences have a similar median error is unknown, but some error is expected between point of crime and point of reporting. Besides geographical uncertainty, there is the problem of using crime statistics in general. For example, the problems of underreporting or narcotic offences being correlated to police resource allocation (Black, 1970) complicated the interpretation of these statistics.

Finally, the use of demographic data and crime statistics offers only one perspective. It cannot tap into the dynamics of the places. For example, which actors commonly attend the places and how their behaviour is perceived by or affect others (such as residents or business owners). One way to add such a perspective could be to conduct key informant interviews. Prior research shows that it is an efficient way to gain reliable information about disorder (Hardyns et al., 2021; Pauwels & Hardyns, 2009) and could be a valuable venue for further research to learn more about the characteristics of identified ODS in smaller communities.

## Conclusions

Despite these challenges, the present study provides important insights into the patterns and characteristics of identified ODS across municipalities of varying sizes. Practitioners consistently identify crime-burdened places in their communities in need of crime prevention efforts because the identified ODS are often associated with lower SES and elevated crime rates. However, the extent of criminal activity varies widely, particularly in smaller municipalities. For this reason, practitioners should be cautious in applying the term “ODS” and reserve it only for places that meet the definition discussed in this study.

The variability in criminal activity across different locations underscores the complexity of addressing drug-related issues and highlights the need for nuanced, location-specific approaches to crime prevention and community safety.
